# OpenSAFELY: factors associated with COVID-19 death in 17 million patients

**DOI:** 10.1038/s41586-020-2521-4

**Published:** 2020-07-08

**Authors:** Elizabeth J Williamson, Alex J Walker, Krishnan Bhaskaran, Seb Bacon, Chris Bates, Caroline E Morton, Helen J Curtis, Amir Mehrkar, David Evans, Peter Inglesby, Jonathan Cockburn, Helen I McDonald, Brian MacKenna, Laurie Tomlinson, Ian J Douglas, Christopher T Rentsch, Rohini Mathur, Angel YS Wong, Richard Grieve, David Harrison, Harriet Forbes, Anna Schultze, Richard Croker, John Parry, Frank Hester, Sam Harper, Rafael Perera, Stephen JW Evans, Liam Smeeth, Ben Goldacre

**Affiliations:** 1 The DataLab, Nuffield Department of Primary Care Health Sciences, University of Oxford, OX26GG; 2 London School of Hygiene and Tropical Medicine, Keppel Street, London WC1E 7HT; 3 TPP, TPP House, 129 Low Lane, Horsforth, Leeds, LS18 5PX; 4 ICNARC, 24 High Holborn, Holborn, London WC1V 6AZ; 5 NIHR Health Protection Research Unit (HPRU) in Immunisation

**Keywords:** COVID-19, risk factors, ethnicity, deprivation, death, informatics

## Abstract

COVID-19 has rapidly impacted on mortality worldwide.^1^ There is unprecedented urgency to understand who is most at risk of severe outcomes, requiring new approaches for timely analysis of large datasets.

Working on behalf of NHS England we created OpenSAFELY: a secure health analytics platform covering 40% of all patients in England, holding patient data within the existing data centre of a major primary care electronic health records vendor. Primary care records of 17,278,392 adults were pseudonymously linked to 10,926 COVID-19 related deaths.

COVID-19 related death was associated with: being male (hazard ratio 1.59, 95%CI 1.53-1.65); older age and deprivation (both with a strong gradient); diabetes; severe asthma; and various other medical conditions. Compared to people with white ethnicity, black and South Asian people were at higher risk even after adjustment for other factors (HR 1.48, 1.29-1.69 and 1.45, 1.32-1.58 respectively).

We have quantified a range of clinical risk factors for COVID-19 related death in the largest cohort study conducted by any country to date. OpenSAFELY is rapidly adding further patients’ records; we will update and extend results regularly.

## Introduction

On March 11th 2020, the World Health Organisation characterised COVID-19 as a pandemic after 118,000 cases and 4,291 deaths were reported in 114 countries.^2^ As of 6 May (the date of latest data availability for this study), cases reached over 3.5 million globally, with more than 240,000 deaths attributed to the virus.1 On the same day in the UK, there were 206,715 confirmed cases, with 30,615 deaths.^3^

Age and gender are well-established risk factors for severe COVID-19 outcomes, with over 90% of UK deaths being in people over 60, and 60% in men^4^. Various pre-existing conditions have also been associated with increased risk. For example, the Chinese center for disease control and prevention (44,672 patients, 1,023 deaths) reported cardiovascular disease, hypertension, diabetes, respiratory disease, and cancers as associated with increased risk of death^5^, but correction for relationships with age was not possible. A UK cross-sectional survey describing 16,749 patients hospitalised with COVID-19 showed higher risk of death for patients with cardiac, pulmonary and kidney disease, as well as malignancy, dementia and obesity (hazard ratios 1.19-1.39 after age and sex correction).^6^ Obesity was associated with treatment escalation in a French ITU cohort (n=124) and a New York hospital presentation cohort (n=3615).^7,8^ Risks associated with smoking are unclear.^9,10,11^ People from black and minority ethnic (BME) groups are at increased risk of bad outcomes from COVID-19, for reasons that are unclear.^12,13^

Patient care is typically managed through electronic health records (EHR) which are commonly used in research. However traditional approaches to EHR analysis rely on intermittent extracts of small samples of historic data. Evaluating a rapidly arising novel cause of death requires a new approach. We therefore set out to deliver a secure analytics platform inside the data centre of major electronic health records vendors, running across the full live linked pseudonymised electronic health records of a very large population of NHS patients, to determine factors associated with COVID-19 related death in England (referred to as “death” in text that follows).

## Results

17,278,392 adults were included (Figure 1; cohort description in Table 1). 1,851,868 (11%) individuals had non-white ethnicities recorded. There were missing data for body mass index (3,751,769, 22%), smoking status (720,923, 4%), ethnicity (4,560,113, 26%), and blood pressure (1,715,095, 10%). 10,926 of the study population had COVID-19 related death recorded in linked death registration data.

The overall cumulative incidence of death 90 days after study start was <0.01% in those aged 18-39 years, rising to 0.67% and 0.44% in men and women respectively aged ≥80 years (Figure 2).

Associations between patient-level factors and risk of death are shown in Table 2 and Figure 3. Increasing age was strongly associated with risk, with those ≥80 years having more than 20-fold increased risk than 50-59 year olds (fully adjusted HR 20.60; 95% CI 18.70-22.68). With age fitted as a flexible spline, an approximately log-linear relationship was observed (Extended Data Figure 1). Men had higher risk than women (fully adjusted HR 1.59, 1.53-1.65). These findings are consistent with patterns observed in smaller studies worldwide and in the UK.^14^

All non-white ethnic groups had higher risk than those with white ethnicity: HRs adjusted for age and sex only ranged from 1.62-1.88 for Black, South Asian and mixed ethnicities compared to white; attenuated to 1.43-1.48 on adjustment for all included risk factors (results for more detailed categories are shown in Extended Data Table 1). Non-white ethnicity has previously been found to be associated with increased COVID-19 infection and poor outcomes.^12,13,15^ Our findings show that only a small part of the excess risk is explained by higher prevalence of medical problems such as cardiovascular disease or diabetes among BME people, or higher deprivation.

We found a consistent pattern of increasing risk with greater deprivation, with the most deprived quintile having a HR of 1.79 compared to the least deprived, consistent with recent national statistics.^16^ Again, very little of this increased risk was explained by pre-existing disease or clinical risk factors, suggesting that other social factors may have an important role.

Increasing risks were seen with increasing obesity (BMI >40 fully adjusted HR 1.92, 95% CI 1.72-2.13), and most comorbidities were associated with higher risk of death, including diabetes (with a greater HR for those with recent HbA1c >= 58 mmol/mol), severe asthma (defined as asthma with recent use of an oral corticosteroid), respiratory disease, chronic heart disease, liver disease, stroke/dementia, other neurological diseases, reduced kidney function (with greater HR for lower estimated glomerular filtration rate), autoimmune diseases (rheumatoid arthritis, lupus or psoriasis) and other immunosuppressive conditions, as per Table 2. Those with a recent (<5 years) history of haematological malignancy had a ≥2.5-fold increased risk, decreasing slightly after 5 years. For other cancers, increased HRs were smaller and mainly with recent diagnoses. History of dialysis or end-stage renal failure was associated with increased risk when added in a secondary analysis (HR 3.69, 3.09-4.39). These findings largely concurred with other data including the UK ISARIC study of hospitalised UK patients with COVID-19 that indicated increased risk of death with cardiac, pulmonary and kidney disease, malignancy, obesity and dementia,^6^ and a large Chinese study which, though lacking age correction, suggested cardiovascular disease, hypertension, diabetes, respiratory disease, and cancers to be associated with increased mortality.^5^ Our findings that severe asthma was associated with higher risk were notable since early data suggested underrepresentation of asthma in patients hospitalised or with severe COVID-19 outcomes,^17,18^

### Post-hoc analyses: smoking and hypertension

Both current and former smoking were associated with higher risk in models adjusted for age and sex only, but in the fully adjusted model current smoking was associated with a lower risk (fully adjusted HR 0.89, CI 0.82-0.97), concurring with lower than expected smoking prevalences in previous studies among hospitalised patients in China,^10^ France^11^ and the USA.^19^ We further explored this post-hoc by adding covariates individually to the age, sex and smoking model, and found the change in HR to be largely driven by adjustment for chronic respiratory disease (HR 0.98, 0.90-1.06 after adjustment). This and other comorbidities could be consequences of smoking, highlighting that the fully adjusted smoking HR cannot be interpreted causally due to the inclusion of factors likely to mediate smoking effects. We therefore then fitted a model adjusted for demographic factors only (age, sex, deprivation, ethnicity), which showed a non-significant positive HR for current smoking (HR 1.07, 0.98-1.18). This does not support any postulated protective effect of nicotine ^9,20^ but suggests that any increased risk with current smoking is likely to be small, and will need to be clarified as the epidemic progresses and more data accumulate.

We similarly explored the change in the hypertension HR (from 1.09, 1.05-1.14 adjusted for age and sex to 0.89, 0.85-0.93 with all covariates included), and found diabetes and obesity to be principally responsible for this reduction (HR 0.97, 0.92-1.01 adjusted for age, sex, diabetes, obesity). Given the strong association between blood pressure and age we then examined an interaction between these variables; this revealed strong evidence of interaction (p<0.001) with hypertension associated with higher risk up to age 70 years and lower risk at older ages (adjusted HRs 3.10 [1.69-5.70], 2.73 [1.96-3.81], 2.07 [1.73-2.47], 1.32 [1.17-1.50], 0.94 [0.86-1.02], 0.73 [0.69-0.78] for ages 18-<40, 40-<50, 50-<60, 60-<70, 70-<80 and >80 respectively). The reasons for the inverse association between hypertension and mortality in older individuals are unclear and warrant further investigation including detailed examination by frailty, comorbidity and drug exposures in this age group.

### Model checking and sensitivity analyses

The average C-statistic was 0.78. Results were similar when missing data were handled using analysis of complete records only, or using multiple imputation (sensitivity analyses: Extended Data Table 2). Non-proportional hazards were detected in the primary model (p<0.001). A sensitivity analysis with earlier administrative censoring at 6th April 2020, before which mortality should not have been affected by UK social distancing policies introduced in late March, showed no evidence of non-proportional hazards (p=0.83). HRs were similar but somewhat larger in magnitude for some covariates, while the association with increasing deprivation appeared to be smaller (Extended Data Table 2).

## Discussion

### Summary

This secure analytics platform operating across over 23 million patient records for the COVID-19 emergency was used to identify, quantify, and explore risk factors for COVID-19 related death in the largest cohort study conducted by any country to date. Most comorbidities were associated with increased risk, including cardiovascular disease, diabetes, respiratory disease including severe asthma, obesity, history of haematological malignancy or recent other cancer, kidney, liver, neurological and autoimmune conditions. People from South Asian and black groups had a substantially higher risk of death, only partially attributable to co-morbidity, deprivation or other risk factors. A strong association between deprivation and risk was only partly attributable to co-morbidity or other risk factors.

These analyses provide a preliminary picture of how key demographic characteristics and a range of comorbidities, a priori selected as being of interest in COVID-19, are jointly associated with poor outcomes. These initial results may be used subsequently to inform the development of prognostic models. We caution against interpreting our estimates as causal effects. For example, the fully adjusted smoking hazard ratio does not capture the causal effect of smoking due to the inclusion of comorbidities which are likely to mediate any effect of smoking on COVID-19 death (e.g. COPD). Our study has highlighted a need for carefully designed causal analyses specifically focusing on the causal effect of smoking on COVID-19 death. Similarly, there is a need for analyses exploring the causal relationships underlying the associations observed between hypertension and COVID-19 death.

### Strengths and weaknesses

The greatest strengths of this study were speed and size. By building a secure analytics platform across routinely collected live clinical data stored in situ we have produced timely results from the current records of approximately 40% of the English population. This scale allows more precision, on rarer exposures, on multiple risk factors, and rapid detection of important signals. Our platform will expand to provide updated analyses over time. Another strength is our use of open methods: we pre-specified our analysis plan and shared our full analytic code and code lists for review and re-use. We ascertained demographics, medications and co-morbidities from full pseudonymised longitudinal primary care records, providing substantially more detail than data recorded on admission, and on the total population rather than the selected subset presenting at hospital. We censored deaths from other causes using ONS data. Analyses were stratified by area to account for known geographical differences in incidence of COVID-19.

We also identify important limitations. In our outcome definition, we included clinically suspected (non laboratory confirmed) COVID-19, because testing has not always been carried out, especially in older patients in care homes. However, this may have incorrectly identified some patients as having COVID-19. Some COVID-19 deaths may have been misclassified as non-COVID-19, particularly in the early stages of the pandemic, though this is likely to have reduced quickly as deaths accumulated, and a degree of outcome underascertainment, providing unrelated to patient characteristics, should not have biased our hazard ratios. Due to the rarity of the outcome, the associations observed will be driven primarily by the profile of risk factors in the included cases. Our findings reflect both an individual’s risk of infection, and their risk of dying once infected. We will explore more detailed patient trajectories in future research within the OpenSAFELY platform.

Our large population may not be fully representative. We include only 17% of general practices in London, where many earlier COVID-19 cases occurred, due to the substantial geographic variation in choice of EHR system.. The user interface of electronic health records can affect prescribing of certain medicines^21–23^ so it is possible that coding may vary between systems.

Primary care records, though detailed and longitudinal, can be incomplete for data on risk factors and other covariates. Ethnicity was missing for approximately 26%, but was broadly representative;^24^ there were also missing data on obesity and smoking. Sensitivity analyses found our estimates were robust to our assumptions around missing data.

Non-proportional hazards could be due to very large numbers or unmeasured covariates. However, rapid changes in social behaviours (social distancing, shielding) and changes in the burden of infection may also have affected patient groups differentially. The larger hazard ratios seen for several covariates in a sensitivity analysis with earlier censoring (soon after social distancing and shielding policies were introduced) are consistent with more at-risk patients being more compliant with these policies. In contrast, the risk associated with deprivation may have increased over time. Subsequent analyses will further explore changes before and after national initiatives around COVID-19.

### Policy Implications and Interpretation

The UK has a policy of recommending shielding (staying at home at all times and avoiding any face to face contact) for groups identified as being extremely vulnerable to COVID-19 on the basis of pre-existing medical conditions.^25^ We were able to evaluate the association between most of these conditions and death from COVID-19, and confirmed increased mortality risks, supporting the targeted use of additional protection measures for people in these groups. We have demonstrated -for the first time -that only a small part of the substantially increased risks of COVID-19 related death among non-white groups and among people living in more deprived areas can be attributed to existing disease. Improved strategies to protect people in these groups are urgently needed.^26^ These might include specific consideration of BME groups in shielding guidelines and work-place policies. Subsequent studies are needed to investigate the interplay of additional factors we were unable to explore, including employment, access to personal protective equipment and related risk of exposure to infection and household density.

The UK has an unusually large volume of very detailed longitudinal patient data, especially through primary care. We believe the UK has a responsibility to the global community to make good use of such data. OpenSAFELY demonstrates at an unprecedented scale that this can be done securely, transparently, and rapidly. We will enhance the OpenSAFELY platform to further inform the global response to the COVID-19 emergency.

### Future Research

The underlying causes of higher risk of COVID-19 related death among those from nonwhite backgrounds, and deprived areas, require further exploration; we would suggest collecting data on occupational exposure and living conditions as first steps. The statistical power offered by our approach means that associations with less common risk factors can be robustly assessed in more detail, at the earliest possible date, as the pandemic progresses. We will therefore update our findings and address smaller risk groups as new cases arise over time. The open source reusable codebase on OpenSAFELY supports rapid, secure and collaborative development of new analyses: we are currently conducting expedited studies on the impact of various medical treatments and population interventions on the risk of COVID-19 infection, ITU admission, and death, alongside other observational analyses. OpenSAFELY is rapidly scalable for additional NHS patients’ records, with new data sources progressing.

### Conclusion

We generated early insights into risk factors for COVID-19 related death using an unprecedented scale of 17 million patients’ detailed primary care records, maintaining privacy, in the context of a global health emergency.

## Methods

### Study design

We conducted a cohort study using national primary care electronic health record data linked to COVID-19 death data (see Data Source). The cohort study began on 1st February 2020, chosen as a date several weeks prior to the first reported COVID-19 deaths and the day after the second laboratory confirmed case;^27^ and ended on 6th May 2020. The cohort explores risk among the general population rather than in a population infected with SARS-COV-2. Therefore, all patients were included irrespective of any SARS-COV-2 test results.

### Data Source

We used patient data from general practice (GP) records managed by the GP software provider The Phoenix Partnership (TPP), linked to Office for National Statistics (ONS) death data. ONS data includes information on all deaths, including COVID-19 related death, defined as a COVID-19 ICD-10 code mentioned anywhere on the death certificate and non-COVID-19 death, which was used for censoring.

The data were accessed, linked and analysed using OpenSAFELY, a new data analytics platform created to address urgent questions relating to the epidemiology and treatment of COVID-19 in England. OpenSAFELY provides a secure software interface that allows detailed pseudonymised primary care patient records to be analysed in near real-time where they already reside, hosted within the EHR vendor’s highly secure data centre, to minimise the re-identification risks when data are transported off-site; other smaller datasets are linked to these data within the same environment using a matching pseudonym derived from the NHS number. More information can be found on https://opensafely.org/.

The dataset analysed with OpenSAFELY is based on 24 million currently registered patients (approximately 40% of the English population) from GP surgeries using the TPP SystmOne electronic health record system. SystmOne is a secure centralised EHR used in English clinical practice since 1998; it records data entered (in real time) by GPs and practice staff during routine primary care. The system is accredited under the NHS approved systems framework for General Practice.^28,29^ Data extracted from TPP SystmOne have previously been used in medical research, as part of the ResearchOne dataset.^30,31^ From this EHR a pseudonymised dataset was created for OpenSAFELY consisting of 20 billion rows of structured data including for example pseudonymised patients’ diagnoses, medications, physiological parameters, and prior investigations [Extended Data Figure 2, Level 1]. All OpenSAFELY data processing took place on TPP’s servers; external data providers securely transferred pseudoymised data (such as COVID-19 related death from ONS) for linkage to OpenSAFELY [Extended Data Figure 2, Level 2]; study definitions developed in Python on GitHub were pulled into the OpenSAFELY infrastructure, and used to create a study dataset of one row per patient [Extended Data Figure 2, Level 3]. Statistical code was developed using synthetic data and used to analyse the study dataset; this included code to check data ranges, to check consistency of data columns, and to produce descriptive statistics for comparison with expected disease prevalences to ensure validity, as well as code to fit our analysis models. Only two authors (KB/AJW) accessed OpenSAFELY to run code; no pseudonymised patient-level data were ever removed from TPP infrastructure; only aggregated, anonymous, manually checked study results were released for publication [Extended Data Figure 2, Level 4], All code for data management and analysis is archived online (see Code Availability, below).

### Study Population and Observation Period

Our study population consisted of all adults (males and females 18 years and above) currently registered as active patients in a TPP general practice in England on 1st February 2020. To be included in the study, participants were required to have at least 1 year of prior follow-up in the GP practice to ensure that baseline patient characteristics could be adequately captured, and to have recorded sex, age, and deprivation (see covariates, below).^32^ Patients were observed from the 1st of February 2020 and were followed until the first of either their death date (whether COVID-19 related or due to other causes) or the study end date, 6th May 2020. For this analysis, ONS death data were available to 11th May 2020, but we used an earlier censor date to allow for delays in reporting in the last few days of available data.

### Outcomes

The outcome was death among people with COVID-19, ascertained from ONS death certificate data, where the COVID related ICD-10 codes U071 or U072 were present in the record.

### Covariates

Potential risk factors included: health conditions listed in UK guidance on “higher risk” groups;^33^ other common conditions which may cause immunodeficiency inherently or through medication (cancer and common autoimmune conditions); and emerging risk factors for severe outcomes among COVID-19 cases (such as raised blood pressure).

Age, sex, body mass index (BMI; kg/m^2^), and smoking status were considered as potential risk factors. Where categorised, age groups were: 18-<40, 40-<50, 50-<60, 60-<70, 70-<80, 80+ years. BMI was ascertained from weight measurements within the last 10 years, restricted to those taken when the patient was over 16 years old. Obesity was grouped using categories derived from the World Health Organisation classification of BMI: no evidence of obesity <30 kg/m^2^; obese I 30-34.9; obese II 35-39.9; obese III 40+. Smoking status was grouped into current, former and never smokers.

The following comorbidities were also considered potential risk factors: asthma, other chronic respiratory disease, chronic heart disease, diabetes mellitus, chronic liver disease, chronic neurological diseases, common autoimmune diseases (Rheumatoid Arthritis (RA), Systemic Lupus Erythematosus (SLE) or psoriasis), solid organ transplant, asplenia, other immunosuppressive conditions, cancer, evidence of reduced kidney function, and raised blood pressure or a diagnosis of hypertension.

Disease groupings followed national guidance on risk of influenza infection,^34^ therefore “chronic respiratory disease (other than asthma)” included COPD, fibrosing lung disease, bronchiectasis or cystic fibrosis; chronic heart disease included chronic heart failure, ischaemic heart disease, and severe valve or congenital heart disease likely to require lifelong follow-up. Chronic neurological conditions were separated into diseases with a likely cardiovascular aetiology (stroke, TIA, dementia) and conditions in which respiratory function may be compromised such as motor neurone disease, myasthenia gravis, multiple sclerosis, Parkinson’s disease, cerebral palsy, quadriplegia or hemiplegia, and progressive cerebellar disease. Asplenia included splenectomy or a spleen dysfunction, including sickle cell disease. Other immunosuppressive conditions included HIV or a condition inducing permanent immunodeficiency ever diagnosed, or aplastic anaemia or temporary immunodeficiency recorded within the last year. Haematological malignancies were considered separately from other cancers to reflect the immunosuppression associated with haematological malignancies and their treatment. Kidney function was ascertained from the most recent serum creatinine measurement, where available, converted into estimated glomerular filtration rate (eGFR) using the Chronic Kidney Disease Epidemiology Collaboration (CKD-EPI) equation,^35^ with reduced kidney function grouped into defined as eGFR 30-<60 or <30 mL/min/1.73m^2^. History of kidney dialysis or end-stage renal failure was separately explored in a secondary analysis. Raised blood pressure (BP) was defined as either a prior coded diagnosis of hypertension or the most recent recording indicating systolic BP ≥140 mmHg or diastolic BP ≥90 mmHg.

Asthma was grouped by use of oral corticosteroids as an indication of severity. Diabetes was grouped according to the most recent Hba1c measurement within the last 15 months (Hba1c <58 mmols/mol, ≥58 mmols/mol, no recent measure available). Cancer was grouped by time since the first diagnosis (within the last year, 2-<5 years, ≥5 years).

Other covariates considered as potential upstream risk factors were deprivation and ethnicity. Deprivation was measured by the Index of Multiple Deprivation (IMD, in quintiles, with higher values indicating greater deprivation), derived from the patient’s postcode at lower super output area level for a high degree of precision. Ethnicity was grouped into White, Black, South Asian, Mixed, or Other. In sensitivity analyses, a more detailed grouping of ethnicity was explored. The Sustainability and Transformation Partnership (STP, an NHS administrative region) of the patient’s general practice was included as an additional adjustment for geographical variation in infection rates across the country.

Information on all covariates were obtained from primary care records by searching TPP SystmOne records for specific coded data. TPP SystmOne allows users to work with the SNOMED-CT clinical terminology, using a GP subset of SNOMED-CT codes. This subset maps on to the native Read version 3 (CTV3) clinical coding system that SystmOne is built on. Medicines are entered or prescribed in a format compliant with the NHS Dictionary of Medicines and Devices (dm+d),^36^ a local UK extension library of SNOMED. Code lists for particular underlying conditions and medicines were compiled from a variety of sources. These include BNF codes from OpenPrescribing.net, published codelists for asthma,^37–39^ immunosuppression,^40–42^ psoriasis,^43^ SLE,^44^ RA^45,46^ and cancer,^47,48^ and Read Code 2 lists designed specifically to describe groups at increased risk of influenza infection.^18^ Read Code 2 lists were added to with SNOMED codes and cross-checked against NHS QOF registers, then translated into CTV3 with manual curation. Decisions on every code list were documented and final lists reviewed by at least two authors. Detailed information on compilation and sources for every individual codelist is available at https://codelists.opensafely.org/ and the lists are available for inspection and re-use by the broader research community.^49^

### Statistical Analysis

Patient numbers are depicted in a flowchart. The Kaplan-Meier failure function was estimated by age group and sex. For each potential risk factor, a Cox proportional hazards model was fitted, with days in study as the timescale, stratified by geographic area (STP), and adjusted for sex and age modelled using restricted cubic splines. Violations of the proportional hazards assumption were explored by testing for a zero slope in the scaled Schoenfeld residuals. All potential risk factors, including age (again modelled as a spline), sex, BMI, smoking, index of multiple deprivation quintile, and comorbidities listed above were then included in a single multivariable Cox proportional hazards model, stratified by STP. Hazard ratios from the age/sex adjusted and fully adjusted models are reported with 95% confidence intervals. Models were also refitted with age group fitted as a categorical variable in order to obtain hazard ratios by age group.

In the primary analysis, those with missing BMI were assumed non-obese and those with missing smoking information were assumed to be non-smokers on the assumption that both obesity and smoking would be likely to be recorded if present. A sensitivity analysis was run among those with complete BMI and smoking data only. Ethnicity was omitted from the main multivariable model due to 26% of individuals having missing data; hazard ratios for ethnicity were therefore obtained from a separate model among individuals with complete ethnicity only. Hazard ratios for other risk factors, adjusted for ethnicity, were also obtained from this model and are presented in the sensitivity analyses to allow assessment of whether estimates may have been distorted by ethnicity in the primary model. We conducted an additional sensitivity analysis using a population-calibrated imputation approach to handle missing ethnicity,^50,51^ with marginal proportions of each ethnicity group within each of nine broad geographical regions of England (East, East Midlands, London, North East, North West, South East, South West, West Midlands, Yorkshire and The Humber) taken from Annual Population Survey (APS) data (pooled 2014-2016).^52^ Five imputed datasets were created with estimated hazard ratios combined using Rubin’s rules.

The C-statistic was calculated as a measure of model discrimination. Due to computational time, this was estimated by randomly sampling 5000 patients with and without the outcome and calculating the C-statistic using the random sample, repeating this 10 times and taking the average C-statistic.

All p-values presented are two-sided.

### Information governance and ethics

NHS England is the data controller; TPP is the data processor; and the key researchers on OpenSAFELY are acting on behalf of NHS England. This implementation of OpenSAFELY is hosted within the TPP environment which is accredited to the ISO 27001 information security standard and is NHS IG Toolkit compliant;^53,54^ patient data has been pseudonymised for analysis and linkage using industry standard cryptographic hashing techniques; all pseudonymised datasets transmitted for linkage onto OpenSAFELY are encrypted; access to the platform is via a virtual private network (VPN) connection, restricted to a small group of researchers, their specific machine and IP address; the researchers hold contracts with NHS England and only access the platform to initiate database queries and statistical models; all database activity is logged; only aggregate statistical outputs leave the platform environment following best practice for anonymisation of results such as statistical disclosure control for low cell counts.^55^ The OpenSAFELY research platform adheres to the data protection principles of the UK Data Protection Act 2018 and the EU General Data Protection Regulation (GDPR) 2016. In March 2020, the Secretary of State for Health and Social Care used powers under the UK Health Service (Control of Patient Information) Regulations 2002 (COPI) to require organisations to process confidential patient information for the purposes of protecting public health, providing healthcare services to the public and monitoring and managing the COVID-19 outbreak and incidents of exposure.^56^ Taken together, these provide the legal bases to link patient datasets on the OpenSAFELY platform. GP practices, from which the primary care data are obtained, are required to share relevant health information to support the public health response to the pandemic, and have been informed of the OpenSAFELY analytics platform. This study was approved by the Health Research Authority (REC reference 20/LO/0651) and by the LSHTM Ethics Board (reference 21863).

## Extended Data

**Figure 1:**
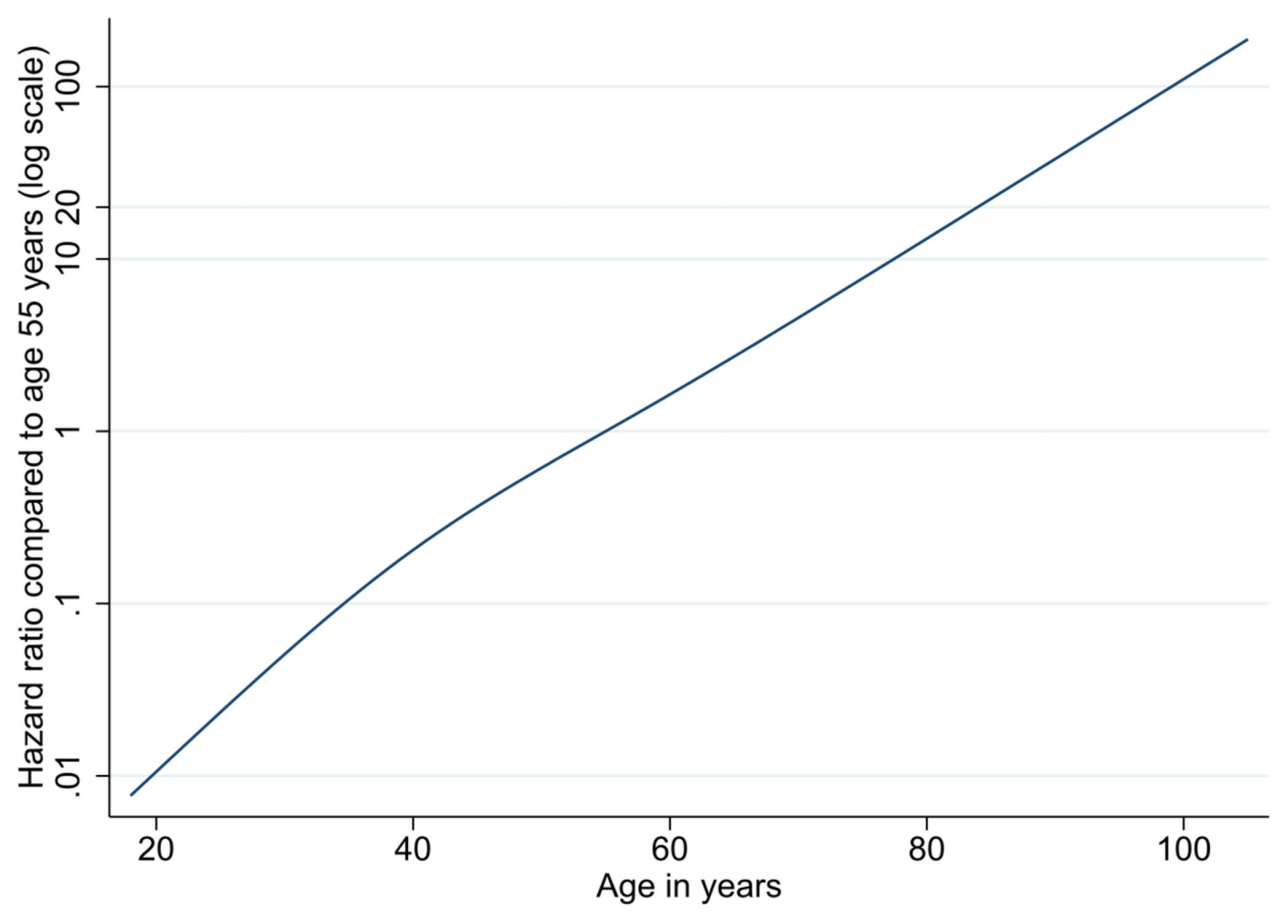
Estimated log hazard ratio by age in years Footnote: From the primary fully adjusted model containing a 4-knot cubic spline for age, and adjusted for all covariates listed in Table 2 except for ethnicity.

**Figure 2:**
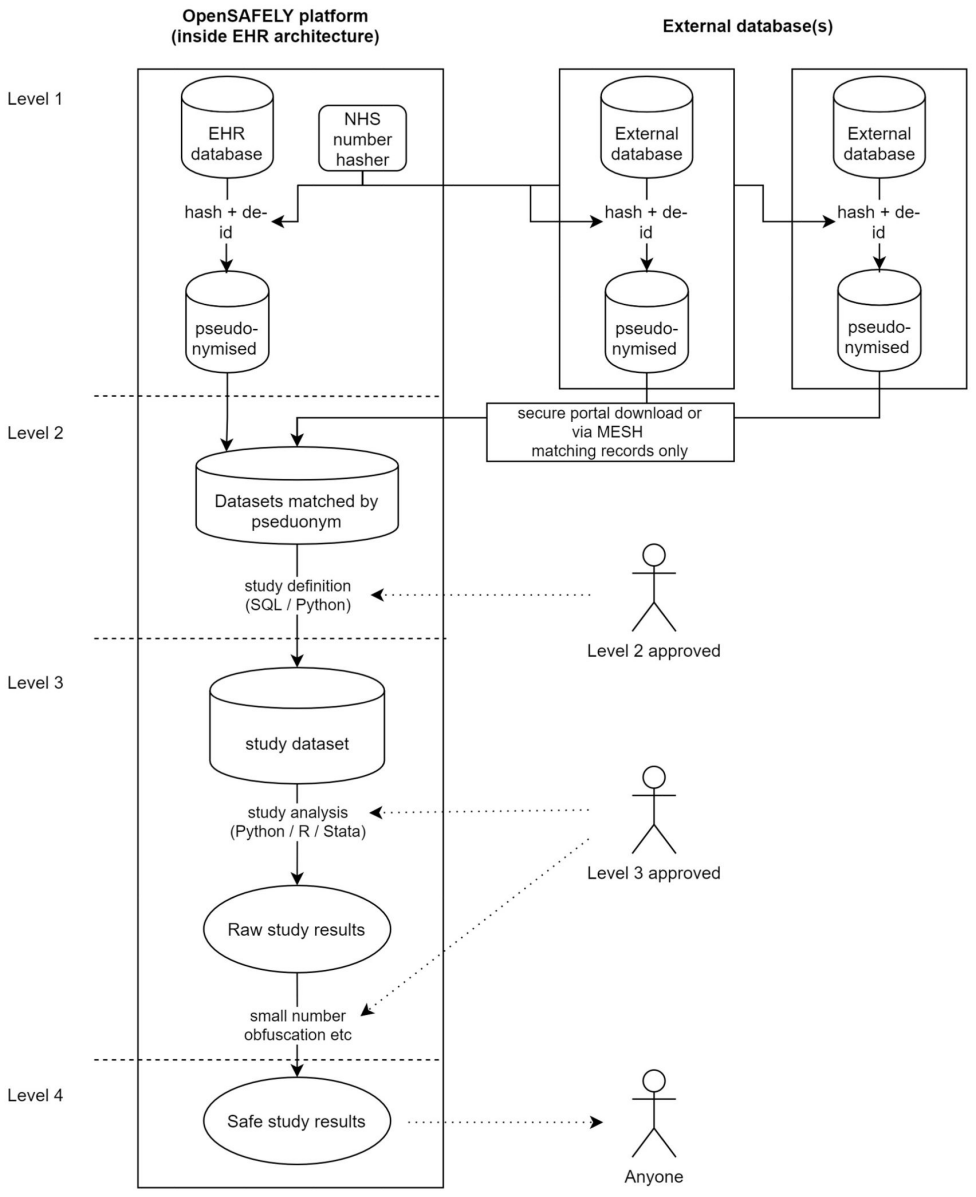
Illustration of data flows in the OpenSAFELY platform

**Extended Data Table 1:** Adjusted hazard ratios for detailed ethnicity categories

Ethnicity	Fully adjusted hazard ratio*	95% CI
British or mixed British	1.00	(ref)
Irish	1.16	(0.96-1.41)
Other White	0.87	(0.79-0.97)
Mixed ethnicity	1.42	(1.11-1.83)
Indian or British Indian	1.40	(1.23-1.59)
Pakistani or British Pakistani	1.24	(1.06-1.46)
Bangladeshi or British Bangladeshi	1.84	(1.36-2.49)
Other Asian	1.73	(1.44-2.09)
Caribbean	1.28	(1.07-1.53)
African	1.77	(1.41-2.22)
Other Black	1.73	(1.24-2.40)
Chinese	1.22	(0.81-1.85)
Other	1.35	(1.09-1.67)

EstEstimated from a model restricted to those with recorded ethnicity, adjusted for age using a 4-knot cubic spline age spline, sex, BMI, smoking, IMD quintile, hypertension/high blood pressure, asthma, chronic heart disease, diabetes, non-haematological cancer, haematological malignancy, reduced kidney function, liver disease, stroke/dementia, other neurological disease, organ transplant, asplenia, rheumatoid/lupus/psoriasis, other immunosuppressive condition; all categorisations are as in the primary analysis.imated from a model restricted to those with recorded ethnicity, adjusted for age using a 4-knot cubic spline age spline, sex, BMI, smoking, IMD quintile, hypertension/high blood pressure, asthma, chronic heart disease, diabetes, non-haematological cancer, haematological malignancy, reduced kidney function, liver disease, stroke/dementia, other neurological disease, organ transplant, asplenia, rheumatoid/lupus/psoriasis, other immunosuppressive condition; all categorisations are as in the primary analysis.

**Extended Data Table 2:** Hazard Ratios (HRs) and 95% confidence intervals (CI) in sensitivity analyses

		Fully adjusted HR and 95% CI				
Characteristic	Category	Primary analysis	Early censoring at 6/4/2020	Restricted to those with complete BMI /smoking	Adjusted for ethnicity in those where recorded	Adjusted for ethnicity using multiple imputation
***N outcome events in analysis***		10926	2816	9880	8149	
**Age**	18-<40	0.06 (0.04-0.08)	0.07 (0.04-0.12)	0.07 (0.05-0.10)	0.07 (0.05-0.09)	0.06 (0.04-0.07)
	40-<50	0.30 (0.25-0.36)	0.33 (0.23-0.46)	0.30 (0.25-0.37)	0.29 (0.24-0.36)	0.29 (0.24-0.35)
	50-<60	1.00 (ref)	1.00 (ref)	1.00 (ref)	1.00 (ref)	1.00 (ref)
	60-<70	2.40 (2.16-2.66)	2.55 (2.11-3.08)	2.38 (2.13-2.66)	2.37 (2.11-2.67)	2.43 (2.19-2.70)
	70-<80	6.07 (5.51-6.69)	5.84 (4.88-6.98)	5.96 (5.37-6.61)	6.05 (5.42-6.76)	6.24 (5.66-6.87)
	80+	20.60 (18.70-22.68)	14.66 (12.23-17.58)	19.96 (18.00-22.14)	20.19 (18.08-22.54)	21.19 (19.23-23.34)
**Sex**	Female	1.00 (ref)	1.00 (ref)	1.00 (ref)	1.00 (ref)	1.00 (ref)
	Male	1.59 (1.53-1.65)	1.89 (1.75-2.05)	1.65 (1.58-1.72)	1.54 (1.47-1.61)	1.57 (1.52-1.64)
**BMI**	Not obese	1.00 (ref)	1.00 (ref)	1.00 (ref)	1.00 (ref)	
	30-34.9kg/m2 (Obese class I)	1.05 (1.00-1.11)	1.30 (1.18-1.43)	1.07 (1.02-1.13)	1.05 (0.99-1.11)	1.06 (1.00-1.11)
	35-39.9kg/m2 (Obese class II)	1.40 (1.30-1.52)	1.57 (1.36-1.81)	1.45 (1.34-1.57)	1.41 (1.30-1.54)	1.42 (1.32-1.54)
	≥40 kg/m2 (Obese class III)	1.92 (1.72-2.13)	2.70 (2.26-3.21)	1.99 (1.79-2.21)	1.92 (1.70-2.17)	1.96 (1.76-2.18)
**Smoking**	Never	1.00 (ref)	1.00 (ref)	1.00 (ref)	1.00 (ref)	1.00 (ref)
	Former	1.19 (1.14-1.24)	1.27 (1.17-1.39)	1.18 (1.13-1.24)	1.22 (1.16-1.29)	1.23 (1.18-1.29)
	Current	0.89 (0.82-0.97)	0.93 (0.79-1.09)	0.91 (0.83-0.99)	0.93 (0.84-1.02)	0.93 (0.85-1.01)
**Ethnicity** *	White	1.00 (ref)	1.00 (ref)	1.00 (ref)	1.00 (ref)	1.00 (ref)
	Mixed	1.43 (1.11-1.84)	1.01 (0.60-1.72)	1.38 (1.05-1.80)	1.43 (1.11-1.84)	1.44 (1.06-1.95)
	South Asian	1.45 (1.32-1.58)	1.63 (1.38-1.91)	1.51 (1.38-1.66)	1.45 (1.32-1.58)	1.48 (1.33-1.65)
	Black	1.48 (1.29-1.69)	1.76 (1.41-2.19)	1.47 (1.28-1.69)	1.48 (1.29-1.69)	1.53 (1.32-1.77)
	Other	1.33 (1.10-1.61)	1.84 (1.37-2.47)	1.40 (1.15-1.71)	1.33 (1.10-1.61)	1.34 (1.12 1.61)
**IMD quintile**	1 (least deprived)	1.00 (ref)	1.00 (ref)	1.00 (ref)	1.00 (ref)	1.00 (ref)
	2	1.12 (1.05-1.19)	0.96 (0.85-1.08)	1.12 (1.05-1.19)	1.16 (1.08-1.25)	1.12 (1.05-1.19)
	3	1.22 (1.15-1.30)	1.00 (0.88-1.12)	1.23 (1.15-1.31)	1.26 (1.17-1.36)	1.21 (1.14-1.29)
	4	1.51 (1.42-1.61)	1.26 (1.11-1.41)	1.51 (1.42-1.61)	1.54 (1.43-1.66)	1.48 (1.39-1.57)
	5 (*most deprived*)	1.79 (1.68-1.91)	1.41 (1.25-1.60)	1.80 (1.68-1.93)	1.77 (1.64-1.91)	1.72 (1.61-1.84)
**Blood pressure**	Normal	1.00 (ref)	1.00 (ref)	1.00 (ref)	1.00 (ref)	1.00 (ref)
	High bp or diagnosed hyper-tension	0.89 (0.85-0.93)	0.95 (0.87-1.04)	0.88 (0.84-0.92)	0.91 (0.86-0.96)	0.89 (0.85-0.93)
**Respiratory disease ex asthma**		1.63 (1.55-1.71)	1.86 (1.69-2.04)	1.59 (1.51-1.67)	1.65 (1.56-1.75)	1.64 (1.56-1.72)
**Asthma (vs none)** **	With no recent OCS use	0.99 (0.93-1.05)	1.08 (0.96-1.20)	0.97 (0.91-1.04)	0.94 (0.87-1.00)	0.98 (0.93-1.05)
	With recent OCS use	1.13 (1.01-1.26)	1.38 (1.13-1.67)	1.09 (0.97-1.22)	1.08 (0.95-1.23)	1.11 (0.99-1.24)
**Chronic heart disease**		1.17 (1.12-1.22)	1.37 (1.26-1.48)	1.16 (1.11-1.22)	1.16 (1.11-1.22)	1.17 (1.12-1.22)
**Diabetes (vs none)** ***	With HbA1c<58 mmol/mol	1.31 (1.24-1.37)	1.39 (1.26-1.52)	1.29 (1.23-1.36)	1.28 (1.21-1.36)	1.27 (1.21-1.33)
	WithHbA1c>=58 mmol/mol	1.95 (1.83-2.08)	2.33 (2.08-2.61)	1.90 (1.78-2.02)	1.86 (1.73-2.00)	1.87 (1.76-1.99)
	With no recent HbA1c measure	1.90 (1.72-2.09)	1.71 (1.40-2.08)	1.92 (1.74-2.12)	1.86 (1.67-2.08)	1.84 (1.67-2.02)
**Cancer (non-haematological, vs none)**	Diagnosed < 1 year ago	1.72 (1.50-1.96)	1.66 (1.27-2.16)	1.68 (1.46-1.94)	1.67 (1.43-1.96)	1.74 (1.52-1.99)
	Diagnosed 1-4.9 years ago	1.15 (1.05-1.27)	1.34 (1.13-1.60)	1.16 (1.05-1.28)	1.21 (1.09-1.35)	1.17 (1.06-1.28)
	Diagnosed ≥5 years ago	0.96 (0.91-1.03)	0.92 (0.81-1.04)	0.97 (0.91-1.03)	0.98 (0.92-1.06)	0.97 (0.92-1.04)
**Haematological malignancy (vs none)**	Diagnosed < 1 year ago	2.80 (2.08-3.78)	2.20 (1.14-4.24)	2.86 (2.10-3.88)	2.33 (1.60-3.41)	2.81 (2.08-3.79)
	Diagnosed 1-4.9 years ago	2.46 (2.06-2.95)	3.49 (2.61-4.68)	2.40 (1.99-2.90)	2.53 (2.05-3.11)	2.48 (2.07-2.97)
	Diagnosed ≥5 years ago	1.61 (1.39-1.87)	1.45 (1.06-1.97)	1.61 (1.38-1.89)	1.55 (1.30-1.85)	1.63 (1.40-1.89)
**Reduced kidney function** ****	Estimated GFR 30-60	1.33 (1.28-1.40)	1.49 (1.36-1.63)	1.33 (1.27-1.39)	1.37 (1.30-1.44)	1.33 (1.27-1.39)
	Estimated GFR <30	2.52 (2.33-2.72)	2.98 (2.57-3.45)	2.47 (2.28-2.68)	2.50 (2.29-2.74)	2.50 (2.31-2.70)
**Liver disease**		1.75 (1.51-2.03)	1.92 (1.48-2.49)	1.69 (1.44-1.97)	1.75 (1.48-2.07)	1.75 (1.51-2.03)
**Stroke/dementia**		2.16 (2.06-2.27)	1.74 (1.58-1.93)	2.12 (2.01-2.22)	2.16 (2.05-2.28)	2.16 (2.06-2.27)
**Other neurological disease**		2.58 (2.38-2.79)	2.26 (1.91-2.68)	2.50 (2.30-2.73)	2.53 (2.31-2.77)	2.58 (2.38-2.80)
**Organ transplant**		3.53 (2.77-4.49)	2.55 (1.59-4.10)	3.70 (2.89-4.73)	3.45 (2.62-4.54)	3.48 (2.74-4.44)
**Asplenia**		1.34 (0.98-1.83)	1.87 (1.12-3.11)	1.29 (0.93-1.80)	1.34 (0.94-1.92)	1.33 (0.98-1.82)
**Rheumatoid/Lupus/Psoriasis**		1.19 (1.11-1.27)	1.29 (1.14-1.46)	1.17 (1.09-1.25)	1.15 (1.07-1.24)	1.20 (1.12-1.28)
**Other immunosuppressive condition**		2.21 (1.68-2.90)	2.60 (1.65-4.09)	2.11 (1.58-2.83)	2.24 (1.66-3.03)	1.67 (1.31-2.11)

FOOTNOTES: Models adjusted for age using a 4-knot cubic spline age spline, except for estimation of age group hazard ratios.

* Ethnicity hazard ratios in primary analysis estimated from a model restricted to those with recorded ethnicity.

** OCS = oral corticosteroids. Recent OCS use defined as in the year before baseline.

*** HbAHbA1c classification based on latest.

**** GFR == glomerular filtration rate in ml/min/1.73m2, based on most recent serum creatinine measure

## Figures and Tables

**Figure 1:**
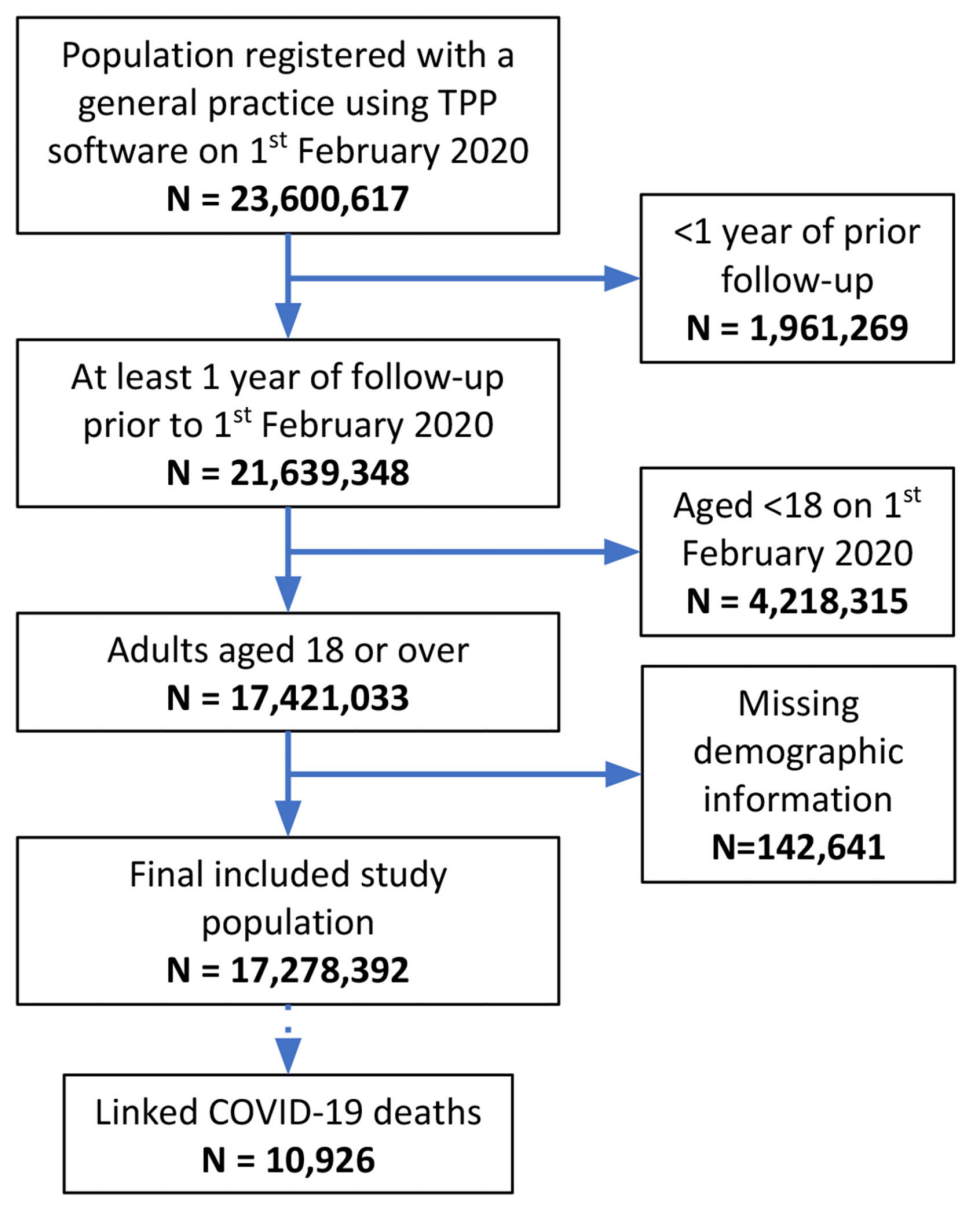
Flow diagram of cohort with numbers excluded at different stages and identification of cases for the main endpoints.

**Figure 2:**
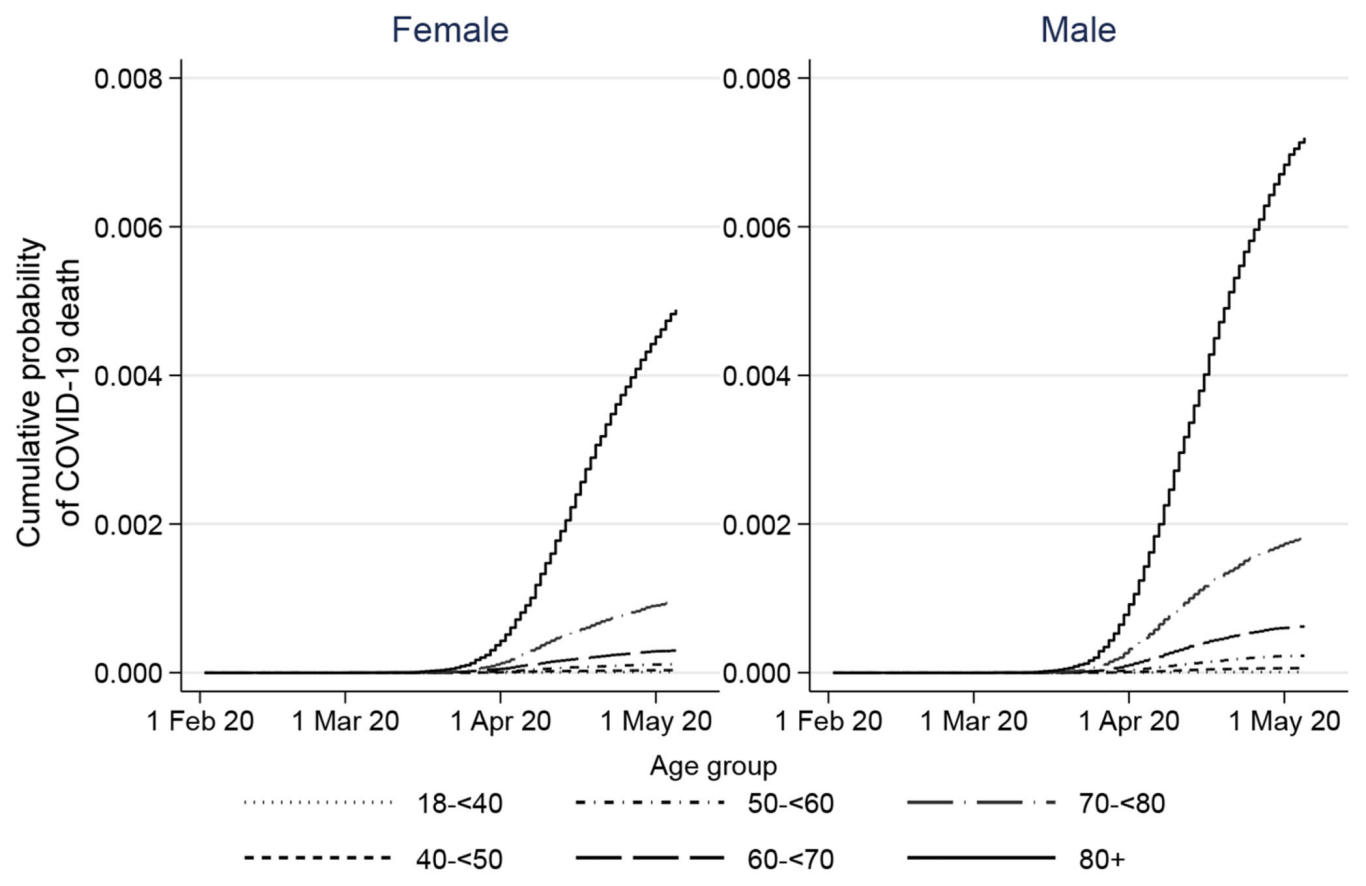
Kaplan-Meier plots for COVID-19 related death over time by age and sex

**Figure 3:**
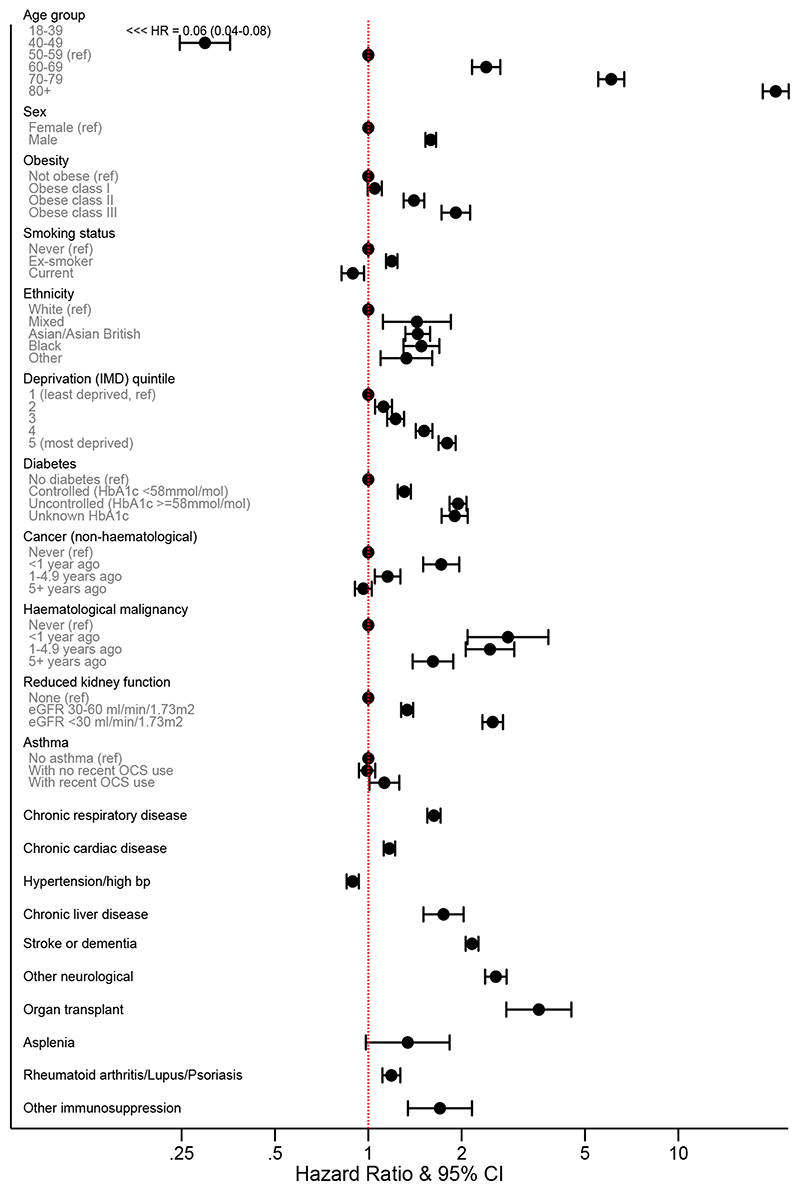
Estimated Hazard Ratios (shown on a log scale) for each potential risk factor from a multivariable Cox model Footnote: Error bars represent limits of the 95% confidence interval for the hazard ratio. Obese class I: 30-34.9kg/m^2^, class II: 35-39.9kg/m^2^, class III: >=40kg/m^2^. OCS = oral corticosteroid. All HRs are adjusted for all other factors listed other than ethnicity. Ethnicity estimates are from a separate model among those with complete ethnicity data, and are fully adjusted for other covariates. Total n = 17,278,392 for non-ethnicity models, and 12,718,279 for ethnicity model.

**Table 1:** Cohort description with number of COVID-19 deaths by potential risk factors

Characteristic	Category	N (column %)	Number of COVID-19 deaths (% within stratum)
**Total**		17,278,392 (100.0)	10,926 (0.06)
**Age**	18-<40	5,914,384 (34.2)	54 (0.00)
	40-<50	2,849,984 (16.5)	140 (0.00)
	50-<60	3,051,110 (17.7)	522 (0.02)
	60-<70	2,392,392 (13.8)	1,101 (0.05)
	70-<80	1,938,842 (11.2)	2,635 (0.14)
	80+	1,131,680 (6.5)	6,474 (0.57)
**Sex**	Female	8,647,989 (50.1)	4,764 (0.06)
	Male	8,630,403 (49.9)	6,162 (0.07)
**BMI (kg/m2)**	<18.5	310,721 (1.8)	522 (0.17)
	18.5-24.9	4,763,150 (27.6)	3,364 (0.07)
	25-29.9	4,682,906 (27.1)	3,068 (0.07)
	30-34.9 (Obese class I)	2,384,406 (13.8)	1,813 (0.08)
	35-39.9 (Obese class II)	922,398 (5.3)	762 (0.08)
	£40 (Obese class III)	463,042 (2.7)	379 (0.08)
	Missing	3,751,769 (21.7)	1,018 (0.03)
**Smoking**	Never	7,924,739 (45.9)	3,598 (0.05)
	Former	5,690,966 (32.9)	6,531 (0.11)
	Current	2,941,764 (17.0)	708 (0.02)
	Missing	720,923 (4.2)	89 (0.01)
**Ethnicity**	White	10,866,411 (62.9)	7,119 (0.07)
	Mixed	169,697 (1.0)	62 (0.04)
	South Asian	1,022,130 (5.9)	608 (0.06)
	Black	339,909 (2.0)	250 (0.07)
	Other	320,132 (1.9)	110 (0.03)
	Missing	4,560,113 (26.4)	2,777 (0.06)
**IMD quintile**	1 (least deprived)	3,497,154 (20.2)	1,908 (0.05)
	2	3,476,668 (20.1)	2,030 (0.06)
	3	3,483,668 (20.2)	2,114 (0.06)
	4	3,480,459 (20.1)	2,388 (0.07)
	5 (most deprived)	3,340,443 (19.3)	2,486 (0.07)
**Blood pressure**	Normal	3,804,148 (22.0)	2,487 (0.07)
	Elevated	2,482,710 (14.4)	1,899 (0.08)
	High Stage 1	5,548,198 (32.1)	3,281 (0.06)
	High Stage 2	3,728,241 (21.6)	3,229 (0.09)
	Missing	1,715,095 (9.9)	30 (0.00)
**High bp or diagnosed hypertension**		5,925,492 (34.3)	8,049 (0.14)
**Respiratory disease ex asthma**		703,917 (4.1)	2,240 (0.32)
Asthma*	With no recent ocs use	2,454,403 (14.2)	1,211 (0.05)
	With recent ocs use	291,670 (1.7)	335 (0.11)
**Chronic heart disease**		1,167,455 (6.8)	3,811 (0.33)
Diabetes**	With HbA1c<58 mmol/mol	1,038,082 (6.0)	2,391 (0.23)
	With HbA1c>=58 mmol/mol	486,491 (2.8)	1,254 (0.26)
	With no recent HbA1c measure	193,993 (1.1)	444 (0.23)
**Cancer (nonhaematological)**	Diagnosed < 1 year ago	79,964 (0.5)	220 (0.28)
	Diagnosed 1-4.9 years ago	234,186 (1.4)	449 (0.19)
	Diagnosed £5 years ago	542,320 (3.1)	1,125 (0.21)
**Haematological malignancy**	Diagnosed < 1 year ago	8,704 (0.1)	43 (0.49)
	Diagnosed 1-4.9 years ago	27,742 (0.2)	120 (0.43)
	Diagnosed £5 years ago	63,460 (0.4)	173 (0.27)
Reduced kidney function***	Estimated GFR 30-60	1,007,383 (5.8)	3,987 (0.40)
	Estimated GFR <30	78,093 (0.5)	864 (1.11)
**Kidney dialysis**		23,978 (0.1)	192 (0.80)
**Liver disease**		100,017 (0.6)	181 (0.18)
**Stroke/dementia**		390,002 (2.3)	2,423 (0.62)
**Other neurological disease**		170,448 (1.0)	665 (0.39)
**Organ transplant**		20,001 (0.1)	69 (0.34)
**Asplenia**		27,917 (0.2)	40 (0.14)
**Rheumatoid/Lupus/ Psoriasis**		878,475 (5.1)	962 (0.11)
**Other immunosuppressive condition**		44,504 (0.3)	52 (0.12)

* ocs= oral corticosteroid use, recent is <1 year before baseline,

** classification by HbAlc based on measures within 15 months before baseline,

*** GFR= glomerular filtration rate (ml/min/1.73m2), from most recent serum creatinine measure

**Table 2:** Hazard Ratios (HRs) and 95% confidence intervals (CI) for COVID-19 death

Characteristic	Category	COVID-19 Death HR (95% CI)	
		Age-sex adj	Fully adj
**Age**	18-<40	0.05 (0.04-0.07)	0.06 (0.04-0.08)
	40-<50	0.28 (0.23-0.33)	0.30 (0.25-0.36)
	50-<60	1.00 (ref)	1.00 (ref)
	60-<70	2.79 (2.52-3.10)	2.40 (2.16-2.66)
	70-<80	8.62 (7.84-9.46)	6.07 (5.51-6.69)
	80+	38.29 (35.02-41.87)	20.60 (18.70-22.68)
**Sex**	Female	1.00 (ref)	1.00 (ref)
	Male	1.78 (1.71-1.85)	1.59 (1.53-1.65)
**BMI**	Not obese	1.00 (ref)	1.00 (ref)
	30-34.9kg/m2 (Obese class I)	1.23 (1.17-1.30)	1.05 (1.00-1.11)
	35-39.9kg/m2 (Obese class II)	1.81 (1.68-1.95)	1.40 (1.30-1.52)
	>40 kg/m2 (Obese class III)	2.66 (2.39-2.95)	1.92 (1.72-2.13)
**Smoking**	Never	1.00 (ref)	1.00 (ref)
	Former	1.43 (1.37-1.49)	1.19 (1.14-1.24)
	Current	1.14 (1.05-1.23)	0.89 (0.82-0.97)
Ethnicity*	White	1.00 (ref)	1.00 (ref)
	Mixed	1.62 (1.26-2.08)	1.43 (1.11-1.84)
	South Asian	1.69 (1.54-1.84)	1.45 (1.32-1.58)
	Black	1.88 (1.65-2.14)	1.48 (1.29-1.69)
	Other	1.37 (1.13-1.65)	1.33 (1.10-1.61)
**IMD quintile**	1 (least deprived)	1.00 (ref)	1.00 (ref)
	2	1.16 (1.08-1.23)	1.12 (1.05-1.19)
	3	1.31 (1.23-1.40)	1.22 (1.15-1.30)
	4	1.69 (1.59-1.79)	1.51 (1.42-1.61)
	5 (most deprived)	2.11 (1.98-2.25)	1.79 (1.68-1.91)
**Blood pressure**	Normal	1.00 (ref)	1.00 (ref)
	High bp or diagnosed hypertension	1.09 (1.05-1.14)	0.89 (0.85-0.93)
**Respiratory disease ex asthma**		1.95 (1.86-2.04)	1.63 (1.55-1.71)
Asthma (vs none)**	With no recent OCS use	1.13 (1.07-1.20)	0.99 (0.93-1.05)
	With recent OCS use	1.55 (1.39-1.73)	1.13 (1.01-1.26)
**Chronic heart disease**		1.57 (1.51-1.64)	1.17 (1.12-1.22)
Diabetes (vs none)***	With HbA1c<58 mmol/mol	1.58 (1.51-1.66)	1.31 (1.24-1.37)
	With HbA1c>=58 mmol/mol	2.61 (2.46-2.77)	1.95 (1.83-2.08)
	With no recent HbA1c measure	2.27 (2.06-2.50)	1.90 (1.72-2.09)
			
**Cancer (non-haematological, vs none)**	Diagnosed < 1 year ago	1.81 (1.58-2.07)	1.72 (1.50-1.96)
	Diagnosed 1-4.9 years ago	1.20 (1.10-1.32)	1.15 (1.05-1.27)
	Diagnosed >5 years ago	0.99 (0.93-1.06)	0.96 (0.91-1.03)
**Haematological malignancy (vs none)**	Diagnosed < 1 year ago	3.02 (2.24-4.08)	2.80 (2.08-3.78)
	Diagnosed 1-4.9 years ago	2.56 (2.14-3.06)	2.46 (2.06-2.95)
	Diagnosed >5 years ago	1.70 (1.46-1.98)	1.61 (1.39-1.87)
Reduced kidney function (vs none)****	Estimated GFR 30-60	1.56 (1.49-1.63)	1.33 (1.28-1.40)
	Estimated GFR <30	3.48 (3.23-3.75)	2.52 (2.33-2.72)
**Liver disease**		2.39 (2.06-2.77)	1.75 (1.51-2.03)
**Stroke/dementia**		2.57 (2.46-2.70)	2.16 (2.06-2.27)
**Other neurological disease**		3.08 (2.85-3.33)	2.58 (2.38-2.79)
**Organ transplant**		6.00 (4.73-7.61)	3.53 (2.77-4.49)
**Asplen ia**		1.62 (1.19-2.21)	1.34 (0.98-1.83)
**Rheumatoid/Lupus/ Psoriasis**		1.30 (1.21-1.38)	1.19 (1.11-1.27)
**Other immunosuppressive condition**		2.75 (2.10-3.62)	2.21 (1.68-2.90)

Models adjusted for age using a 4-knot cubic spline age spline, except for estimation of age group hazard ratios.

* Ethnicity hazard ratios estimated from a model restricted to those with recorded ethnicity.

** OCS = oral corticosteroids. Recent OCS use defined as in the year before baseline.

*** HbA1c classification based on latest measure within 15 months before baseline.

**** GFR = glomerular filtration rate in ml/min/1.73m2, based on most recent serum creatinine measure

## Data Availability

All data were linked, stored and analysed securely within the OpenSAFELY platform https://opensafely.org/. All code is shared openly for review and re-use under MIT open license. Detailed pseudonymised patient data is potentially re-identifiable and therefore not shared. We rapidly delivered the OpenSAFELY data analysis platform without prior funding to deliver timely analyses on urgent research questions in the context of the global Covid-19 health emergency: now that the platform is established we are developing a formal process for external users to request access in collaboration with NHS England; details of this process will be published shortly on OpenSAFELY.org.

## Code Availability

Data management was performed using Python 3.8 and SQL, with analysis carried out using Stata 16.1 / Python. All code for data management and analysis is archived online at https://github.com/opensafely/risk-factors-research. All clinical and medicines codelists are openly available for inspection and reuse at https://codelists.opensafely.org/.
